# A Phase III Randomized Controlled Trial of Plitidepsin, a Marine-Derived Compound, in Hospitalized Adults With Moderate COVID-19

**DOI:** 10.1093/cid/ciae227

**Published:** 2024-08-26

**Authors:** Pedro Landete, Olga-Adriana Caliman-Sturdza, Jose A Lopez-Martin, Liliana Preotescu, Mihaela-Catalina Luca, Anastasia Kotanidou, Paula Villares, Shirley-Patricia Iglesias, Pablo Guisado-Vasco, Elena-Maria Saiz-Lou, Maria del Carmen Farinas-Alvarez, Esperanza Merino de Lucas, Eduardo Perez-Alba, Jose-Miguel Cisneros, Vicente Estrada, Carmen Hidalgo-Tenorio, Garyfallia Poulakou, Miguel Torralba, Jesus Fortun, Paula Garcia-Ocana, Adrien Lemaignen, Miguel Marcos-Martin, Maria Molina, Roger Paredes, Maria Teresa Perez-Rodriguez, Dimitar Raev, Pablo Ryan, Fernanda Meira, Javier Gomez, Nadia Torres, Diego Lopez-Mendoza, Jose Jimeno, Jose-Felipe Varona

**Affiliations:** Pneumology Department, Hospital Universitario La Princesa, Madrid, Spain; Research Laboratory, Instituto de Investigación La Princesa (IIS Princesa), Madrid, Spain; Department of Pneumology, Hospital Enfermera Isabel Zendal, Madrid SARS CoV2 Unit, Madrid, Spain; Department of Pneumology, Universidad Autónoma de Madrid, Madrid, Spain; Department of Infectious Diseases, Judetean de Urgenta "Sf. Ioan cel Nou", Suceava, Romania; Deparment of Internal Medicine, University of Suceava, Suceava, Romania; Virology Unit, PharmaMar, Madrid, Spain; Department of Internal Mecicine, Institutul National De Boli Infectioase "Prof. Dr. Matei Bals", Bucharest, Romania; Department of Internal Mecicine, University of Medicine and Pharmacy "Carol Davila", Bucharest, Romania; Department of Internal Medicine, Spitalul Clinic De Boli Infecţioase "Sf. Paraschev", Iasi, Romania; Department of Internal Medicine, "Grigore T. Popa" University, Iasi, Romania; Department of Internal Medicine, National and Kapodistrian University of Athens, Athens, Greece; Pulmonary and Critical Care, Evagelismos General Hospital, Athens, Greece; Internal Medicine, Hospital Universitario HM Sanchinarro, HM Hospitales Group, Madrid, Spain; Infectiology Department, Clinica de la Costa, Barranquilla, Colombia; Internal Medicine, Hospital Universitario Quironsalud Madrid, Madrid, Spain; Medical Research Center, Universidad Europea, Madrid, Spain; Virology Unit, PharmaMar, Madrid, Spain; Infectious Diseases Department, Hospital Universitario ‘Marqués de Valdecilla’, Santander, Spain; Department of Internal Medicine, Valdecilla Research Institute (IDIVAL), Santander, Spain; Department of Medicine and Psychiatry, Universidad de Cantabria, Research Center, Santander, Spain; Unit of Infectious Diseases, Alicante General University Hospital, Alicante, Spain; Department of Infectious Disease, Alicante Institute of Health and Biomedical Research (ISABIAL), Alicante, Spain; Infectology Department, Hospital Universitario "Dr. José Eleuterio González", Monterrey, Mexico; Department of Infectious Diseases, Universidad Autónoma de Nuevo León, Monterrey, Mexico; Department of Research, Institute of Biomedicine of Seville (IBiS), Seville, Spain; Department of Infectious Diseases, Virgen del Rocío’ University Hospital, Seville, Spain; Infectious Diseases Unit, Hospital Universitario "San Carlos", Madrid, Spain; Infectious Diseases Unit, Hospital Universitario "Virgen de las Nieves", Granada, Spain; 3rd Department of Internal Medicine and Laboratory, National Sotiria General Hospital, Athens, Greece; Internal Medicine, Guadalajara University Hospital, Guadalajara, Spain; Infectious Diseases Department, Hospital Universitario Ramon y Cajal, Madrid, Spain; Infectious Diseases Unit, Hospital Universitario de Jerez de la Frontera, Cádiz, Spain; Department of Infectious Diseases, Centre Hospitalier Regional et Universitaire de Tours (CHRU Tours)—Hopital Bretonneaut, Tours, France; Department of Medicine, Hospital Universitario de Salamanca, Salamanca, Spain; ILD Unit-Respiratory Department, University Hospital of Bellvitge, Barcelona, Spain; Bellvitge Institute for Biomedical Reseach, IDIBELL, Barcelona, Spain; Department of Research Center, CIBERES, Barcelona, Spain; Infectious Diseases Department, Hospital Universitari Germans Trial I Pujol, Badalona, Spain; Department of Infectious Diseases, IrsiCaixa AIDS Research Institute, Badalona, Spain; Infectious Diseases Unit, Internal Medicine Department, Complexo Hospitalario Universitario de Vigo . Vigo, Spain; Cardiology and Internal Medicine, Internal Medicine Clinic, University Hospital UMHAT “Sveta Anna”, Sofia, Bulgaria; Infectious Diseases Hospital Infanta Leonor, Madrid, Spain; Virology Unit, PharmaMar, Madrid, Spain; Department of Biostatistics, PharmaMar, Madrid, Spain; Department of Data Management, PharmaMar, Madrid, Spain; Virology Unit, PharmaMar, Madrid, Spain; Virology Unit, PharmaMar, Madrid, Spain; Department of Internal Medicine, Hospital Universitario HM Monteprincipe, HM Hospitales, Madrid, Spain; Facultad HM de Ciencias de la Salud, Universidad Camilo Jose Cela, Madrid, Spain

**Keywords:** plitidepsin, SARS-CoV-2, COVID-19, antiviral agents, marine compounds

## Abstract

**Background:**

Plitidepsin has shown potent preclinical activity against severe acute respiratory syndrome coronavirus 2 and was generally well tolerated in a phase I trial of hospitalized patients with coronavirus disease 2019 (COVID-19). NEPTUNO, a phase III, multicenter, randomized, controlled trial, was designed to evaluate the efficacy and safety of plitidepsin in the management of moderate COVID-19 in hospitalized adult patients.

**Methods:**

Included patients had documented severe acute respiratory syndrome coronavirus 2 infection, required oxygen therapy, and had adequate organ function. The planned sample size was 609 patients. Patients were randomized 1:1:1 to at least 3 days of dexamethasone plus either plitidepsin (1.5 mg/day or 2.5 mg/day, for 3 days) or standard of care (control). The primary endpoint was the time to sustained withdrawal of supplemental oxygen. Secondary endpoints included time to sustained hospital discharge, clinical status, duration of oxygen support, percentage of patients requiring admission to the intensive care unit, and safety.

**Results:**

After randomizing 205 patients, NEPTUNO was discontinued due to a notable drop in COVID-19–related hospitalizations. Available data suggest a 2-day improvement in the median time to sustained oxygen therapy discontinuation (5 vs 7 days) favoring both plitidepsin arms (hazard ratio, 1.37; 95% confidence interval, .96–1.96; *P* = .08 for plitidepsin 1.5 mg vs control; hazard ratio, 1.06; 95% confidence interval, .73–1.53; *P* = .78 for plitidepsin 2.5 mg vs control). Plitidepsin was generally well tolerated.

**Conclusions:**

Despite the trial limitations, these results suggest that plitidepsin may have a positive benefit-risk ratio in the management of patients requiring oxygen therapy. Further studies with plitidepsin, including those in immunosuppressed patients, are warranted.

Results from this phase III trial suggest that plitidepsin, a first-in-class antiviral, may have a positive benefit-risk ratio in the management of hospitalized patients requiring oxygen therapy for moderate COVID-19.

Plitidepsin, a cyclic depsipeptide derived from the Mediterranean tunicate *Aplidium albicans* that targets host elongation factor 1a [1], has shown potent antiviral activity against severe acute respiratory syndrome coronavirus 2 (SARS-CoV-2), preventing assembly of viral structures in infected cells, and reducing viral load of several variants of concern in mouse models [2–5]. The phase I APLICOV-PC trial subsequently showed that plitidepsin was generally well tolerated in hospitalized adult patients with coronavirus disease 2019 (COVID-19) [2]. Importantly, plitidepsin's antiviral effects were underscored by an average 3.25–log_10_ reduction in baseline viral load and a high hospital discharge rate (82%) by day 15 [2]. Together, these data justified the initiation of the NEPTUNO trial (NCT04784559, EudraCT 2020-005951-19), described here, which was designed to assess the efficacy and tolerability of 2 dose levels of plitidepsin versus local standard of care in adults with moderate COVID-19 requiring hospitalization and oxygen supplementation.

## METHODS

### Study Design

This multicenter, open-label, controlled phase III trial (EudraCT Number: 2020-005951-19; NCT04784559) involved patients enrolled between June 2021 and January 2023 at 28 sites in 8 countries (Supplementary Table 1). The protocol and amendments (see Supplementary Material) were approved by the ethics committees or institutional review boards at each participating site. The trial was designed and conducted in accordance with the Declaration of Helsinki and its most recent update and the International Council for Harmonization E6 Good Clinical Practices guideline. All participants provided written informed consent before initiating protocol-specific procedures.

### Participants

Hospitalized patients older than age 18 years were eligible for inclusion if they had documented SARS-CoV-2 infection by either qualitative polymerase chain reaction (PCR) or antigen test from samples collected no more than 72 hours before study treatment on day 1; a maximum of 14 days from symptom onset; requirement for oxygen therapy (category 5 on the 11-point World Health Organization [WHO] Clinical Progression Scale) [6]; and adequate bone marrow, liver, kidney, and metabolic function. Patients could be included in this trial having had already received a small molecule treatment (eg, remdesivir, molnupiravir, nirmatrelvir/ritonavir) if said treatment was given at least 24 hours previously, outside a clinical trial, and there was documentation of objective clinical deterioration plus evidence of persisting positivity for SARS-CoV-2 in appropriate biological samples. Key exclusion criteria included having prebaseline (ie, in the month that preceded the current SARS-CoV-2 infection) impairment in general health condition for whatever reason except COVID-19; requiring severe dependency for daily living activities (Barthel index <60/100) [7] or chronic oxygen therapy; evidence of respiratory failure at the time of randomization; severe COVID-19 (>category 5 on the 11-point WHO Clinical Progression Scale) [6]; or concomitant antiviral, immunomodulatory, or immunosuppressive therapies at the time of randomization, except for glucocorticoids for a maximum of 72 hours. A complete description of the inclusion and exclusion criteria are provided in the Supplementary Material.

### Randomization and Trial Regimens

Eligible patients were randomly assigned 1:1:1 to receive plitidepsin 1.5 mg/day intravenously (IV), plitidepsin 2.5 mg/day IV, or standard of care. Central randomization was implemented on days 0 to 1 before the first infusion of study drug using stratified permuted blocks to balance groups for stratification factors. Plitidepsin was administered as 1-hour IV infusions on days 1–3 of the trial. Patients in the control group, in accordance with local treatment guidelines, could have received a regulatory-approved antiviral treatment such as remdesivir (200 mg IV on day 1 followed by 100 mg/day IV on days 2–5) or favipiravir (1600 mg twice daily orally on day 1, followed by 600 mg twice daily orally daily for 2–5 days). Randomization was stratified for geographical region (Europe vs rest of the world); age-adjusted Charlson Comorbidity Index (0 to 1 vs >1) [8]; and Barthel Index (≥90 vs <90). (7)

All patients received dexamethasone phosphate 8 mg/day IV (equivalent to a 6.6-mg dexamethasone base) on days 1–3 (administered as a premedication in the plitidepsin arms), followed by dexamethasone base 6 mg/day orally/IV from day 4 and up to a total cumulative dose of 60 mg of dexamethasone base as per physician judgment.

To prevent plitidepsin-induced infusion reactions and emesis, patients received the following premedications: palonosetron 0.25 mg IV; diphenhydramine hydrochloride 25 mg IV (or equivalent such as dexchlorpheniramine maleate 5 mg); and ranitidine 50 mg IV (or equivalent, such as famotidine 20 mg IV).

From treatment initiation on day 1, patients were followed in the hospital for at least 4 days and then through day 31 (±3 days) or resolution/stabilization of treatment-related adverse events (TEAEs), treatment-emergent adverse events of special interest (AESIs), and serious adverse events (SAEs). Patients discharged from the hospital before day 8 returned to an outpatient clinic for assessments on days 8 (±1 day) and 31 (±3 days). On day 15, patients were followed up in remote or onsite visits.

### Trial Endpoints

The primary efficacy endpoint was the time to sustained withdrawal of supplementary oxygen (≤category 4 on the 11-point WHO Clinical Progression Scale) with no subsequent reutilization during remaining study period. The original primary efficacy endpoint of this trial was the complete recovery rate by day 8, defined as meeting categories 0–2 on the 11-point WHO Clinical Progression Scale, having a Barthel index >90/100 at the time of discharge, and not being readmitted to hospital for COVID-19–related signs or symptoms through day 31.

To optimize accrual, the protocol was amended to allow the inclusion of patients with preexisting moderate physical dependence (Barthel score ≥ 60). This necessitated the previously mentioned modification of the primary efficacy endpoint. The sponsor was blinded to the data and the protocol was amended before any data analysis was performed.

The key secondary efficacy endpoint was time to sustained (ie, with no subsequent readmission to day 31) hospital discharge. Other secondary efficacy endpoints included clinical status, as assessed by the 11-category WHO Clinical Progression Scale, at day 8; total duration of advanced oxygen support (high-flow nasal oxygen, extracorporeal membrane oxygenation, noninvasive ventilation or mechanical ventilation); percentage of patients requiring admission to intensive care unit by days 4, 8, 15, and 31; change in the SARS-CoV-2 viral load from day 1 before administration of the study drug until day 8; and percentage of patients with undetectable viral load of SARS-CoV-2 on day 8. Viral load from nasopharyngeal samples was assessed at a central laboratory, using an approved reverse transcriptase-PCR assay.

Safety endpoints included the frequency of TEAEs, frequency of TEAEs of ≥ grade 3 according to National Cancer Institute Common Terminology Criteria for Adverse Events (version 5.0), AESIs, SAEs, drug-related SAEs (ie, serious adverse reactions), adverse events leading to treatment discontinuation, deaths, change from baseline in individual trial-defined laboratory parameters, change from baseline in individual vital signs, and change from baseline in individual trial-defined electrocardiogram parameters.

### Statistical Analysis

This trial was designed to provide at least 80% power to detect a hazard ratio (HR) of 1.4 in the time to sustained withdrawal of supplementary oxygen, equivalent to a decrease in the median time to the event from 8 days (control arm) to 5.7 days (plitidepsin), based on a 1-sided type I error rate of 1.25%. For the trial to have 80% power, a total sample size of 609 patients (203 in each arm) were needed with a minimum of 530 primary endpoint events required to have occurred. Sample size was calculated using a Bonferroni correction for the multiple comparisons of each plitidepsin arm with control arm, although the Hochberg step-up procedure was used for the main analysis, increasing the power of the tests. At the final analysis, if an observed critical HR was >1.27, in favor of any plitidepsin arm (equivalent to a decrease in the median time to sustained withdrawal of supplementary oxygen of >1.7 days), it was expected that the null hypothesis (ie, HR ≤1) was rejected.

Continuous variables were summarized in terms of number of patients/observations with nonmissing data (n), mean, standard deviation, median, interquartile range (25th and 75th percentiles), and range (minimum and maximum). Categorical variables were summarized as frequency counts and associated percentages or difference in percentages. All significance tests were 2-sided and used a 0.05 α-level unless specified otherwise.

Analysis of the primary and other time-to-event efficacy endpoints were performed in the intention-to-treat population. Other secondary efficacy endpoints were based on the full analysis set population, defined as the set of all randomized patients who received ≥1 dose of any study treatment during the trial and had ≥1 postbaseline clinical status collected. Safety analyses were performed on the “as treated” population (patients who received any exposure to study treatments, according to the therapy they actually received). The full statistical analysis plan can be found in section 8 of the trial protocol (see Supplementary Material). Data processing, summarization, and analyses were performed using SAS Environment/version 9.

## RESULTS

### Premature Termination of the Trial

In January 2023, the sponsor made the decision to prematurely terminate enrollment into the NEPTUNO trial after randomization of 205 patients. Patient accrual was negatively impacted by the reduced need for hospitalization and oxygen support, stemming from the emergence of novel variants, increased vaccine penetrance, and approval of therapies for high-risk populations.

### Patient and Disease Characteristics

**Figure 1.**
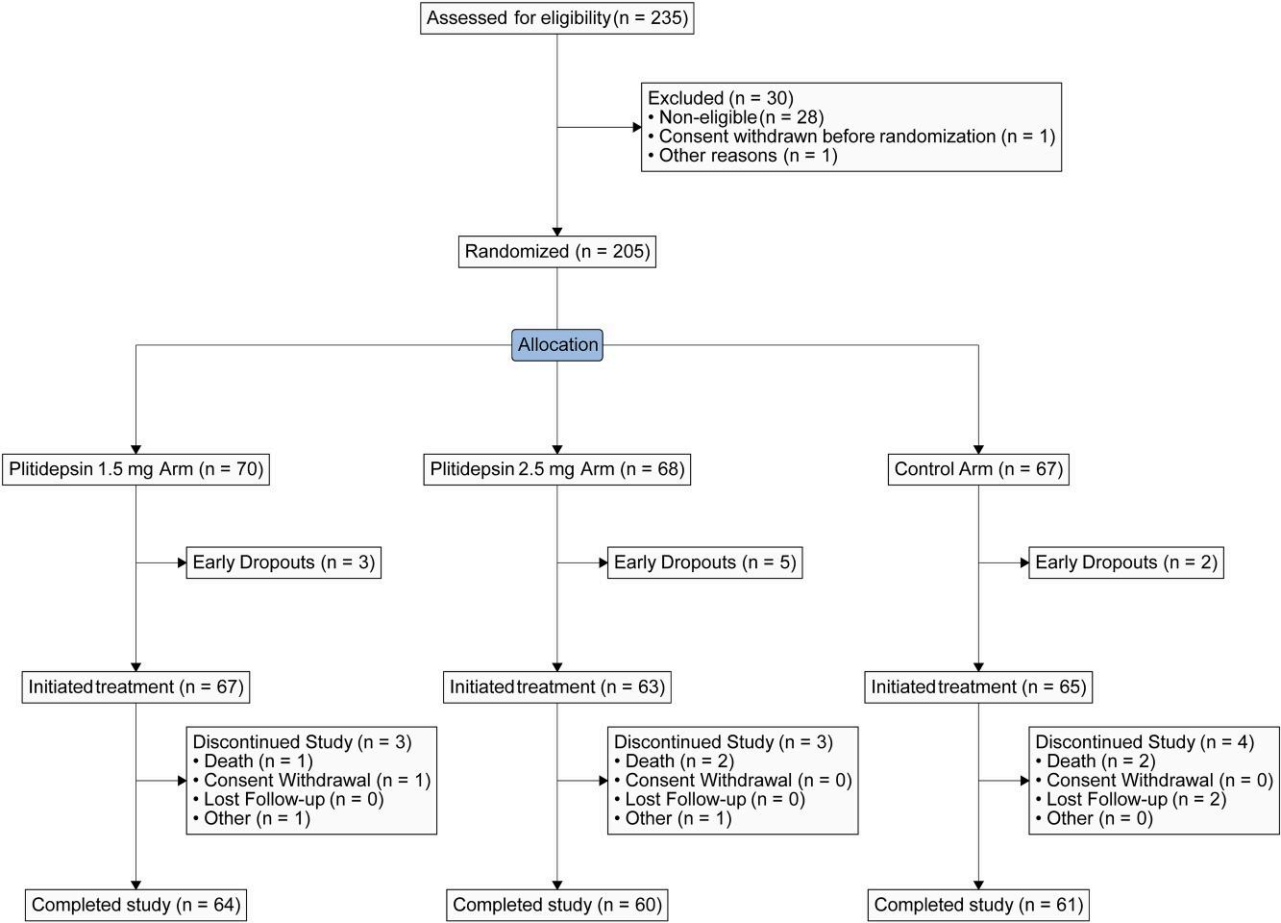
CONSORT diagram.

At the time of trial termination, a total of 235 patients had been screened for this trial across 8 countries (Supplementary Table 1). Of these, 205 patients were randomized to 1 of the 3 treatment arms: 68 to the plitidepsin 2.5-mg arm, 70 to the plitidepsin 1.5-mg arm, and 67 to the control arm. Among these, 10 patients did not receive study treatment (Figure 1). Approximately 90% of randomized patients completed the trial in each arm. Baseline patient and disease characteristics are shown in Table 1. Overall, 164 patients (80.0%) had at least 1 comorbid condition; details can be found in Supplementary Table 2. Details of concomitant COVID-19 medications administered during the trial can be found in the Supplementary Materials, and baseline characteristics by need for concomitant medications are shown in Supplementary Table 3.

**Table 1.** Baseline Patient and Disease Characteristics of the Intention-to-Treat Population

Characteristic Statistics	Plitidepsin 2.5 mg (N = 68)	Plitidepsin 1.5 mg (N = 70)	Control Arm (N = 67)	Total (N = 205)
Age (y)				
n	68	70	67	205
Mean (SD)	58.9 (13.3)	58.1 (14.8)	59.3 (15.0)	58.7 (14.3)
Age group (y), n (%)				
≥18 to 64	45 (66.2)	43 (61.5)	38 (56.7)	126 (61.5)
≥65–74	17 (25.0)	19 (27.1)	15 (22.4)	51 (24.8)
≥75	6 (8.8)	8 (11.5)	14 (20.9)	28 (13.7)
Sex, n (%)				
Male	43 (63.2)	44 (62.9)	42 (62.7)	129 (62.9)
Female	25 (36.8)	26 (37.1)	25 (37.3)	76 (37.1)
Race, n (%)^a^				
Asian	1 (1.5)	3 (4.3)	0	4 (2.0)
White	63 (92.6)	62 (88.6)	64 (95.5)	189 (92.2)
Multiple	4 (5.9)	5 (7.1)	3 (4.5)	12 (5.9)
Body mass index at screening (kg/m^2^)^b^				
N	66	67	65	198
Mean (SD)	29.5 (4.6)	29.7 (5.6)	30.3 (6.1)	29.8 (5.4)
Body mass index group at screening (kg/m^2^)^b^, n (%)				
≥18.5 and <25	10 (14.7)	10 (14.3)	10 (14.9)	30 (14.6)
≥25 and <30	31 (45.6)	35 (50.0)	26 (38.8)	92 (44.9)
≥30 and <35	15 (22.1)	13 (18.6)	17 (25.4)	45 (22.0)
≥35 and <40	9 (13.2)	4 (5.7)	8 (11.9)	21 (10.2)
≥40	1 (1.5)	5 (7.1)	4 (6.0)	10 (4.9)
Missing	2 (2.9)	3 (4.3)	2 (3.0)	7 (3.4)
Chest imaging at enrollment, n (%)				
Pulmonary infiltrates	49 (72.1)	44 (62.9)	43 (64.2)	136 (66.3)
Bilateral pneumonia	20 (29.4)	26 (37.1)	19 (28.4)	65 (31.7)
Periods of inclusion, n (%)				
Beginning of accrual—August 2021	2 (2.9)	1 (1.4)	2 (3.0)	5 (2.4)
September 2021–March 2022	56 (82.4)	55 (78.6)	51 (76.1)	162 (79.0)
April 2022–end of accrual	10 (14.7)	14 (20.0)	14 (20.9)	38 (18.5)
Time from symptom onset to treatment initiation				
N	62	66	65	193
Mean (SD)	5.3 (2.2)	5.3 (2.1)	5.6 (2.3)	5.4 (2.2)
Respiration rate (breaths/min) at screening				
N	68	69	65	202
Mean (SD)	19.5 (3.5)	19.0 (3.4)	19.9 (3.3)	19.4 (3.4)
Oxygen saturation (%) at screening^c^				
N	68	69	67	204
Mean (SD)	96.3 (1.8)	96.3 (1.7)	96.4 (1.7)	96.3 (1.7)
Fraction of inspired oxygen (%) at screening				
N	68	70	67	205
Mean (SD)	26.9 (3.9)	26.9 (3.4)	27.9 (9.5)	27.3 (6.2)
Vaccination status, n (%)				
Fully vaccinated	35 (51.5)	36 (51.4)	32 (47.8)	103 (50.2)
Non-fully vaccinated	6 (8.8)	3 (4.3)	4 (6.0)	13 (6.3)
Not vaccinated	27 (39.7)	31 (44.3)	31 (46.3)	89 (43.4)
S1 spike protein IgG at day 1				
N	55	60	59	174
Negative	19 (34.5)	19 (31.7)	24 (40.7)	62 (35.6)
Positive	36 (65.5)	40 (66.7)	35 (59.3)	111 (63.8)
Borderline	0	1 (1.7)	0	1 (0.6)
SARS-CoV-2 viral load at day 1 (log_10_ copies/mL)^d^				
N	47	56	55	…
Mean (SD)	5.02 (1.88)	4.97 (1.99)	5.06 (2.25)	…

Abbreviations: IgG, immunoglobulin G; n, number of patients with data available; N, number of patients in analysis set; SARS-CoV-2, severe acute respiratory syndrome coronavirus 2; SD, standard deviation; %, percentages are calculated based on N as the denominator.

^a^Patients with more than 1 race reported were included in the multiple categories.

^b^Body mass index (kg/m^2^) = weight (kg)/height (m^2^).

^c^O_2_ at baseline estimated at ambient air with a correction for altitude. The specific modality (mask or nasal prongs) was not collected.

^d^Summary was based on full analysis set.

### Primary Efficacy Endpoint

**Figure 2.**
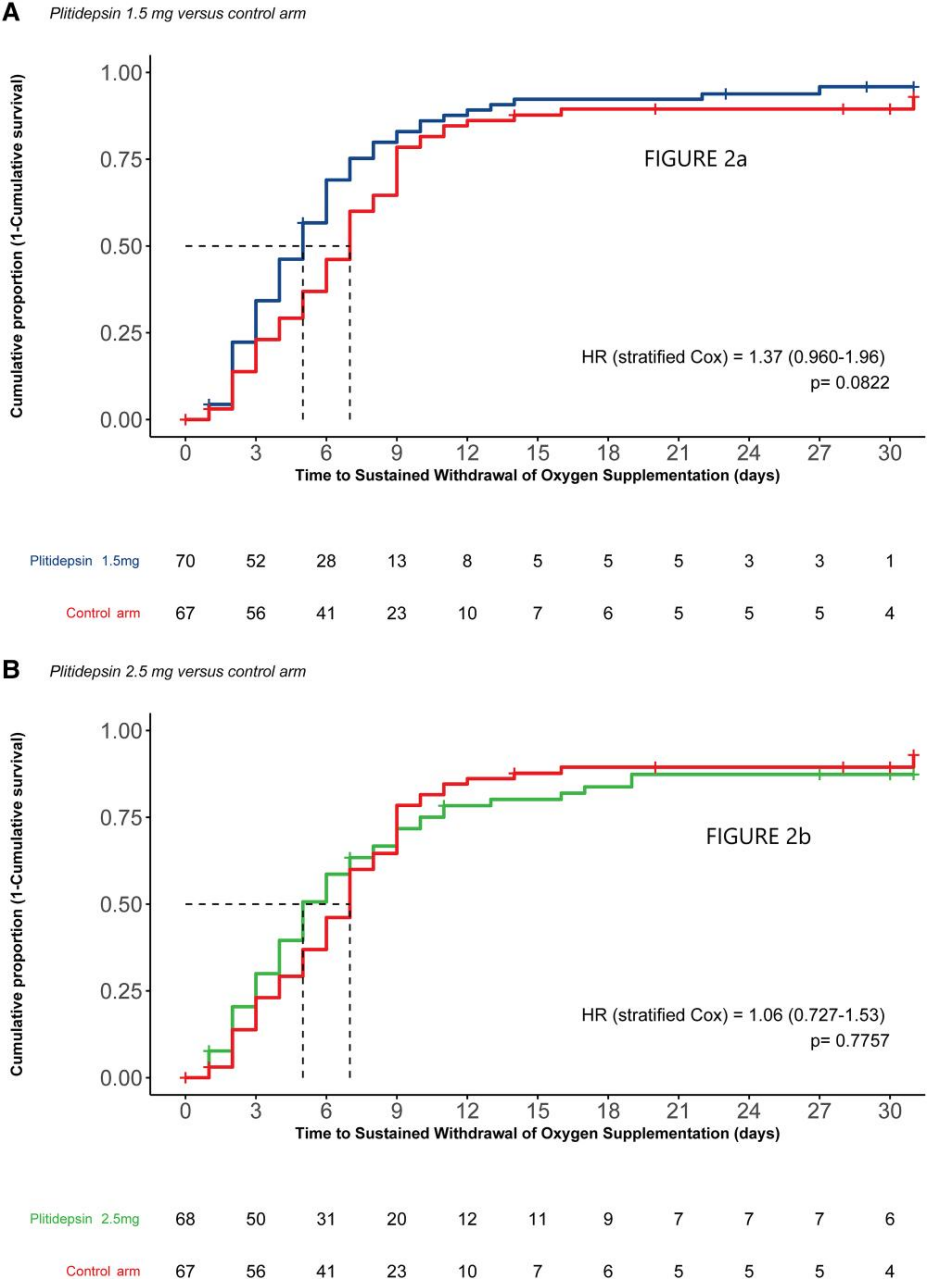
Time to sustained withdrawal of oxygen supplementation for (*A*) plitidepsin 1.5 mg versus control and (*B*) plitidepsin 2.5 mg versus control. Sustained withdrawal of oxygen supplementation (in days) with no subsequent reutilization during remaining study period is defined as the first day, from randomization through completion of the trial, on which a patient satisfies categories 0–4 on the 11-point World Health Organization (WHO) Clinical Progression Scale and has no subsequent reutilization of oxygen supplementation (5–10 on the 11-point WHO Clinical Progression Scale). If the patient is discontinued between day 15 and day 31 visits, and there is an attempt of contact and trial discontinuation on or after the day 31 visit due date, the patient is censored at day 31.

The total number of patients who had sustained withdrawal of oxygen supplementation in the treatment arms were 54 (79.4%), 63 (90.0%), and 59 (88.1%) for patients in the plitidepsin 2.5-mg, plitidepsin 1.5-mg, and control arms, respectively. The Kaplan-Meier estimates of median time to sustained withdrawal of oxygen supplementation were 5 days (95% confidence interval [CI], 4–7), 5 days (95% CI, 4–6) and 7 days (95% CI, 6–8) for the plitidepsin 2.5-mg, plitidepsin 1.5-mg, and control arms, respectively (Figure 2). Stratified log-rank test *P* values for the comparison of time to sustained withdrawal of oxygen supplementation were calculated for plitidepsin 2.5 mg versus control (*P* = .88) and plitidepsin 1.5 mg versus control (*P* = .063), with respective multiplicity-adjusted 2-sided *P* values of *P* = .88 and *P* = .13, respectively.

Point estimated HRs by stratified Cox proportional hazards regression model analysis were >1 for both plitidepsin arms (HR = 1.06 [95% CI, .727–1.53], 2-sided *P* = .78 for plitidepsin 2.5 mg vs control; HR = 1.37 [95% CI, .960–1.96], 2-sided *P* = .082 for plitidepsin 1.5 mg vs control), directionally favoring plitidepsin treatment. Details of a bootstrap analysis providing estimates of the HR and CI from a simulation of the originally planned sample size can be found in the Supplementary Materials.

### Secondary Efficacy Endpoints

The total number of patients reporting sustained hospital discharge was 55 (80.9%), 61 (87.1%), and 59 (88.1%) for patients in the plitidepsin 2.5-mg, plitidepsin 1.5-mg, and control arms, respectively. The Kaplan-Meier estimates of median time to sustained hospital discharge were 7 days (95% CI, 7–9), 7 days (95% CI, not estimated), and 7 days (95% CI, 7–9) for the plitidepsin 2.5-mg, plitidepsin 1.5-mg, and control arms, respectively (Supplementary Figure 1). Stratified log-rank test *P* values for the comparison of time to sustained hospital discharge were calculated for plitidepsin 2.5 mg versus control (*P* = .59) and plitidepsin 1.5 mg versus control (*P* = .34).

For the comparison of plitidepsin 2.5 mg versus control, the stratified Cox proportional hazards regression model estimated an HR = 0.948 (95% CI, .655–1.37) with a 2-sided *P* value of .78. For the comparison of plitidepsin 1.5 mg versus control, an HR = 1.18 (95% CI, .827–1.70) with a 2-sided *P* value of .35. Other secondary endpoints are summarized in Supplementary Table 4.

### Safety and Tolerability

Patients in the plitidepsin 2.5-mg arm (71.4%) had a numerically higher incidence of TEAEs from any cause than patients in the plitidepsin 1.5-mg (65.7%) and control arms (61.5%). Similar observations were noted for incidence of grade ≥3 TEAEs (34.9%, 25.4%, and 16.9% for plitidepsin 2.5 mg, plitidepsin 1.5 mg, and control, respectively) and any-grade treatment-related TEAEs (54.0%, 44.8%, and 36.9% for plitidepsin 2.5 mg, plitidepsin 1.5 mg, and control, respectively) (Table 2; Supplementary Table 5). Hyperglycemia was the most frequent in all arms (Table 3; Supplementary Table 6), and in all cases it was related to dexamethasone. The most frequent treatment-related TEAE reported in the plitidepsin arms was nausea (8%–9%), which was generally of low intensity, controlled by medication, and of short duration.

**Table 2.** Summary of Adverse Events With >10% Incidence in any Treatment arm

Adverse Events Category Adverse Events Type	Plitidepsin 2.5 mg (N = 63) n (%)	Plitidepsin 1.5 mg (N = 67) n (%)	Plitidepsin Total (N = 130) n (%)	Control Arm (N = 65) n (%)
**Any TEAE**	45 (71.4)	44 (65.7)	89 (68.5)	40 (61.5)
Grade ≥3	22 (34.9)	17 (25.4)	39 (30.0)	11 (16.9)
**Any treatment-related TEAE to any study treatment^a^**	34 (54.0)	30 (44.8)	64 (49.2)	24 (36.9)
Grade ≥3	7 (11.1)	6 (9.0)	13 (10.0)	3 (4.6)
**Any serious TEAE**	11 (17.5)	6 (9.0)	17 (13.1)	5 (7.7)
Grade ≥3	10 (15.9)	6 (9.0)	16 (12.3)	5 (7.7)
**System organ class grouped preferred term^a^ (any-cause, all-grade TEAEs)**				
Gastrointestinal disorders	26 (41.3)	20 (29.9)	46 (35.4)	12 (18.5)
Constipation	11 (17.5)	10 (14.9)	21 (16.2)	4 (6.2)
Nausea	9 (14.3)	8 (11.9)	17 (13.1)	1 (1.5)
Diarrhea	9 (14.3)	3 (4.5)	12 (9.2)	3 (4.6)
General disorders and administration site conditions	11 (17.5)	10 (14.9)	21 (16.2)	13 (20.0)
Investigations	23 (36.5)	27 (40.3)	50 (38.5)	19 (29.2)
Serum ferritin abnormal	15 (23.8)	13 (19.4)	28 (21.5)	4 (6.2)
C-reactive protein increased	11 (17.5)	7 (10.4)	18 (13.8)	5 (7.7)
Alanine aminotransferase increased	4 (6.3)	7 (10.4)	11 (8.5)	5 (7.7)
Gamma-glutamyltransferase increased	5 (7.9)	8 (11.9)	13 (10.0)	3 (4.6)
Metabolism and nutrition disorders	22 (34.9)	17 (25.4)	39 (30.0)	12 (18.5)
Hyperglycemia^b^	18 (28.6)	15 (22.3)	33 (25.4)	10 (15.4)
Musculoskeletal and connective tissue disorders	7 (11.1)	6 (9.0)	13 (10.0)	4 (6.2)
Nervous system disorders	8 (12.7)	9 (13.4)	17 (13.1)	6 (9.2)
Headache	6 (9.5)	7 (10.4)	13 (10.0)	5 (7.7)
Psychiatric disorders	13 (20.6)	6 (9.0)	19 (14.6)	5 (7.7)
Sleep disorder	7 (11.1)	6 (9.0)	13 (10.0)	5 (7.7)
Respiratory, thoracic, and mediastinal disorders	17 (27.0)	13 (19.4)	30 (23.1)	13 (20.0)
Acute respiratory distress syndrome	9 (14.3)	8 (11.9)	17 (13.1)	6 (9.2)
Vascular disorders	14 (22.2)	12 (17.9)	26 (20.0)	6 (9.2)
Phlebitis	11 (17.5)	9 (13.4)	20 (15.4)	2 (3.1)

Abbreviations: n, number of patients with the reported adverse event; N, number of patients in analysis set; TEAE, treatment-emergent adverse event; %, percentages are calculated based on N as the denominator.

^a^Within a system organ class, patients could have reported more than 1 grouped preferred term. Patients were counted once for each grouped preferred term and each system organ class.

^b^Hyperglycemia (system organ class = Metabolism and nutrition disorders) also includes Blood glucose increased (system organ class = Investigations).

**Table 3.** Summary of Treatment-Related Adverse Events Reported in > 1 Patient or as Grade ≥ 3

System Organ Class	Preferred Term^a^	Plitidepsin 2.5 mg (N = 63)				Plitidepsin 1.5 mg (N = 67)				Control Arm (N = 65)			
		All		Grade ≥ 3		All		Grade ≥ 3		All		Grade ≥ 3	
		n	%	n	%	n	%	n	%	n	%	n	%
Gastrointestinal disorders	Abdominal pain	1	1.6	…	…	2	3.0	…	…	1	1.5	…	…
	Constipation	3	4.8	…	…	…	…	…	…	1	1.5	…	…
	Diarrhea	4	6.3	…	…	2	3.0	…	…	…	…	…	..
	Nausea	5	7.9	…	…	6	9.0	…	…	…	…	…	…
General disorders	Asthenia	1	1.6	…	…	2	3.0	…	…	…	…	…	…
Infections and infestations	Cellulitis	…	…	…	…	1	1.5	1	1.5	…	…	…	…
Investigations	Adjusted calcium decreased	1	1.6	1	1.6	2	3.0	1	1.5	…	…	…	…
	ALT increased	2	3.2	…	…	3	4.5	…	…	3	4.6	…	…
	AST increased	3	4.8	…	…	1	1.5	…	…	4	6.2	…	…
	CPK increased	…	…	…	…	…	…	…	…	2	3.1	…	…
	Blood LDH increased	…	…	…	…	…	…	…	…	3	4.6	…	…
	γ-GT increased	3	4.8	…	…	3	4.5	…	…	1	1.5	…	…
	Lipase increased	1	1.6	1	1.6	1	1.5	…	…	…	…	…	…
	Serum ferritin abnormal	2	3.2	…	…	2	3.0	…	…	…	…	…	…
Metabolism and nutrition disorders	Hyperglycemia^b^	15	23.8	5	7.0	14	20.9	4	6.0	8	12.3	3	4.6
Vascular disorders	Hypertension	…	…	…	…	2	3.0	…	…	2	3.1	…	…
	Phlebitis	3	4.8	…	…	…	…	…	…	1	1.5	…	…

Abbreviations: γ-GT, gamma transaminases; ALT, alanine liver transaminase; AST, aspartate aminotrasnferase; CPK, creatine phosphokinase; LDH, lactate dehydrogenase; n, number of patients with the reported adverse event; N, number of patients in analysis set; %, percentages are calculated based on N as the denominator.

^a^Within a system organ class, patients could have reported more than 1 grouped preferred term. Patients were counted once for each grouped preferred term and each system organ class.

^b^Hyperglycemia (system organ class = Metabolism and nutrition disorders) also includes Blood glucose increased (system organ class = Investigations).

The incidence of treatment-emergent AESIs of any cause were 34.9%, 37.3%, and 27.7% for plitidepsin 2.5-mg, plitidepsin 1.5-mg, and control groups, respectively. The incidence of SAEs was 17.5%, 9.0%, and 7.7% in the plitidepsin 2.5-mg, plitidepsin 1.5-mg, and control arms, respectively. Fewer than 2% of patients had drug-related SAEs considered to be related to plitidepsin; 1 patient in the 2.5-mg arm had a grade 2 hypersensitivity reaction and 1 patient in the 1.5-mg arm had grade 3 cellulitis.

Fewer than 2% of patients in the treatment arms discontinued any study treatment because of AEs (1 patient in each plitidepsin arm, Figure 1). Five patients died during the trial: 2 each in the plitidepsin 2.5-mg and control arms and 1 in plitidepsin 1.5-mg arm. No fatal events were considered by the investigator as related to study treatment.

## DISCUSSION

Although the trial was terminated early because challenges in accrual, protocol-defined analysis of the available data suggests that plitidepsin may have a positive benefit-risk ratio in the management of patients with COVID-19 who require oxygen therapy.

Across treatment arms, approximately 80% of patients had sustained withdrawal of oxygen supplementation. Although no statistically significant difference between plitidepsin and control arms was observed, the point estimated HRs directionally favored plitidepsin treatment, indicating the potential of a shorter time to sustained withdrawal of oxygen supplementation than the control arm. Notably, for plitidepsin 1.5 mg, the HR of point estimate 1.37 was in line with the targeted HR of the original trial design. The trend toward benefit is corroborated by other endpoints, such as the evaluation of patients’ WHO Clinical Progression Scale, which showed that patients in the plitidepsin arms had numerically lower rates of remaining at a score of 5 by day 8 and were more likely to achieve scores of 0 to 2 versus the control arm. Similarly, patients treated in the plitidepsin arms received a shorter mean duration of corticoid therapy, with a greater number of those on plitidepsin discontinuing corticoids by day 9 compared with the control arm.

Because the trial was ended prematurely, conclusive benefit of plitidepsin could not be confirmed. Accrual for this trial was hindered by several factors related to the evolution of the pandemic. In December 2021, about 6 months after trial initiation, the circulating Delta variant was largely displaced by Omicron [9]. Though Omicron proved more likely to cause breakthrough infections, hospital admissions were less frequent, making recruitment for the trial challenging [9]. Additionally, approval of new antivirals, including nirmatrelvir/ritonavir in the European Union in January 2022, further reduced the pool of high-risk patients infected with SARS-CoV-2 who would need hospitalization [10]. Considering how these developments could be used to mitigate the risk of early trial termination for potential future respiratory pandemics, 1 idea would be to expand the inclusion criteria (eg, to all hospitalized patients requiring supplemental oxygen, regardless of their disease severity) to enlarge the potential pool of participants.

Despite the limited sample size, this trial further supports the tolerability of plitidepsin in patients with moderate COVID-19. As in the previous proof-of-concept trial, most adverse events occurring in this trial were mild and transient in nature [2].

We note that the control arm had a greater proportion of patients who were aged ≥75 years, which is a known risk factor for poorer outcomes and survival [11, 12]. Though considered as a potential covariate that could have influenced the results of the multivariate analysis, Cox regression and random forest models did not find age to be an independent contributor of the time to sustained O_2_ withdrawal. Nevertheless, future studies should consider stratifying by age to ensure equal distributions of this risk factor. Another limitation is that the relative representation of each trial arm per site was not controlled by randomization, and a balanced distribution of the arms locally was limited by the early trial termination and evolution of the pandemic among the different countries. Finally, this trial was not able to identify differences in the kinetics of viral clearance between arms because of the limited number of sampling timepoints and relatively high quantitation limit of the reverse transcriptase-PCR test.

This trial, albeit compromised by the early trial termination, provides important insights on safety and pharmacological impact that makes plitidepsin a rational candidate agent for future pandemics. Ongoing studies are further characterizing the molar potency of plitidepsin to inhibit replication, at noncytotoxic concentrations, in other RNA viruses. The data gathered in dose ranging studies in SARS-CoV-2—particularly the results on safety and posology—will provide a useful foundation for designing future clinical trials.

Given that there remains an unmet need to identify direct-acting antivirals that markedly reduce SARS-CoV-2 replication, plitidepsin could be considered as a candidate therapy for COVID-19 and further studies are warranted. This is particularly important in immunosuppressed patients, in whom compassionate use of plitidepsin has already been shown to be well tolerated with potential clinical and antiviral efficacy [13]. The ongoing phase 2 NEREIDA trial (NCT05705167) will evaluate the efficacy of plitidepsin in prespecified groups of immunocompromised patients with symptomatic COVID-19 requiring hospitalization.

## Supplementary Data

Supplementary materials are available at *Clinical Infectious Diseases* online. Consisting of data provided by the authors to benefit the reader, the posted materials are not copyedited and are the sole responsibility of the authors, so questions or comments should be addressed to the corresponding author.

## Supplementary Material

ciae227_Supplementary_Data

## Notes

***Author contributions.*** O-A. C-S., L. P., M-C. L., A. K., P. V., S-P. I., P. G-V., M-C. F-A, E. M. L., E. P-A., J-M. C., V. E., C. H-T., G. P., M. T., J. F., P. G-O., A. L., M. M-M, M. M., R. P., M. T. P-R., D. R., P. R.: investigation, writing—review. P. L, J. F. V.: investigation, writing—review, validation. D. L-M.: supervision, project administration, and funding acquisition. E-M. S-L.: supervision, validation, and investigation. F. M.: supervision, validation. J. G.: software, methodology, formal analysis and writing—review, visualization. N. T.: software, data curation and validation. J. A. L-M: conceptualization, formal analysis, methodology and writing—original draft, review & editing, and visualization. J. J.: conceptualization, formal analysis, methodology and writing—original draft, review & editing, supervision, and funding acquisition.

***Acknowledgments.*** The authors thank the patients, and their relatives for their participation in this trial and also thank Timothy Silverstein for providing editorial support and Raquel Lloris for dedicated contributions to this trial. Data from this trial are not publicly available.

***Ethical Approval Statement.*** All applicable protocol versions and their respective amendments were evaluated and approved by the Ethics Committees/Institutional Review Boards of the participating sites.

***Financial support*.** This work was supported by PharmaMar SA. (Colmenar Viejo, Spain). Financial support was intended to cover costs derived from the study.
