# Supplementary material for: A Phase III Randomized Controlled Trial of Plitidepsin, a Marine-Derived Compound, in Hospitalized Adults With Moderate COVID-19
Source: Clin Infect Dis. 2024 Aug 26;79(4):910–9. doi: 10.1093/cid/ciae227 (PMC11478586; doi:10.1093/cid/ciae227)
Supplement: ciae227_Supplementary_Data [file ciae227_supplementary_data.zip › NEPTUNO_CID_Supplementary Material.docx]

**Supplementary Material**

**A phase III randomized controlled trial of plitidepsin, a marine derived compound, in hospitalized adults with moderate COVID-19**

Pedro Landete, Olga-Adriana Caliman-Sturdza, Jose A. Lopez-Martin, Liliana Preotescu, Mihaela-Catalina Luca, Anastasia Kotanidou, Paula Villares, Shirley-Patricia Iglesias, Pablo Guisado-Vasco, Elena-Maria Saiz-Lou, Maria del Carmen Farinas-Alvarez, Esperanza Merino de Lucas, Eduardo Perez-Alba, Jose-Miguel Cisneros, Vicente Estrada, Carmen Hidalgo-Tenorio, Garyfallia Poulakou, Miguel Torralba, Jesus Fortun, Paula Garcia-Ocana, Adrien Lemaignen, Miguel Marcos-Martin, Maria Molina, Roger Paredes, Maria Teresa Perez-Rodriguez, Dimitar Raev, Pablo Ryan, Fernanda Meira, Javier Gomez, Nadia Torres, Diego Lopez-Mendoza, Jose Jimeno, Jose-Felipe Varona.

**Supplemental Data**

*Concomitant COVID-19 Medications*

In addition to dexamethasone, remdesivir was given to 28 (43.1%) patients in the control group for a median of 5 days (range 3–5), whereas favipiravir was administered to 8 (12.3%) patients for a median of 5 days (range 2–7). No patient in the plitidepsin arms required remdesivir or favipiravir. The mean (SD) duration of corticoid exposure was 7.8 (2.4) days, 7.7 (2.1) days, and 8.5 (2.2) days for the plitidepsin 2.5 mg, plitidepsin 1.5 mg, and control arms, respectively. A higher proportion of patients in the plitidepsin groups (ranging from 42.9% to 49.3%) received corticoid therapy for less than 9 days during the trial compared to the control group (26.2%). Overall, 16 patients (7.8%) in the trial received immunomodulatory medications. Fewer patients received immunomodulating agents in the plitidepsin 2.5 mg (n=3 [4.4%]) and plitidepsin 1.5 mg (n=3 [4.3%]) arms than in the control arm (n=10 [14.9%]). Each of these patients received a single dose of tocilizumab except 1 patient in the plitidepsin 2.5 mg arm who received 2 doses, and one patient in the control arm who received 1 dose of tocilizumab and 1 dose of anakinra.

*Bootstrap analysis*

In addition to the protocol-defined analyses, the statistical analysis plan was amended at the time of trial termination to include a bootstrap analysis to simulate the originally planned sample size (N = 609) with the current sample size to investigate what would have been the variability of the observed HR and the CI if the trial had been completed as planned. For the comparison of plitidepsin 2.5 mg versus control, the stratified Cox proportional hazards regression model after bootstrap resampling estimated a HR = 1.02 (95% CI: 0.833–1.25) with a 2-sided p = 0.51 adjusted for multiplicity. For the comparison of plitidepsin 1.5 mg versus control, a HR = 1.36 (95% CI: 1.14–1.65) was estimated. When adjusted for multiplicity, plitidepsin 1.5 mg showed a statistically significant difference compared to control (2-sided p = 0.0067).

| **Supplementary Table 1.** Number of Randomized Patients by Country and Site (Intent-To-Treat Population) | | | | | |
| --- | --- | --- | --- | --- | --- |
|  | | | | |  |
| **Country  Site** | **Plitidepsin 2.5mg (N=68) n (%)** | **Plitidepsin 1.5mg (N=70) n (%)** | **Control Arm (N=67) n (%)** | **Total (N=205)  n (%)** |  |
|  | | | | |  |
| **Total** | 68 (100.0) | 70 (100.0) | 67 (100.0) | 205 (100.0) |  |
|  |  |  |  |  |  |
| **Brazil** | 1 (1.5) | 0 | 0 | 1 (0.5) |  |
| BR012 - Hospital Felício Rocho | 1 (1.5) | 0 | 0 | 1 (0.5) |  |
|  |  |  |  |  |  |
| **Bulgaria** | 0 | 0 | 1 (1.5) | 1 (0.5) |  |
| BG018 - “MHAT “Sveta Anna” – Sofia AD | 0 | 0 | 1 (1.5) | 1 (0.5) |  |
|  |  |  |  |  |  |
| **Colombia** | 2 (2.9) | 4 (5.7) | 1 (1.5) | 7 (3.4) |  |
| CO002 - Clínica de la Costa Ltda | 2 (2.9) | 4 (5.7) | 1 (1.5) | 7 (3.4) |  |
|  |  |  |  |  |  |
| **France** | 0 | 1 (1.4) | 0 | 1 (0.5) |  |
| FR018 - CHRU Tours Hopital Bretonneau | 0 | 1 (1.4) | 0 | 1 (0.5) |  |
|  |  |  |  |  |  |
| **Greece** | 6 (8.8) | 4 (5.7) | 6 (9.0) | 16 (7.8) |  |
| GR002 - Sotiria General Hospital Chest Diseases | 1 (1.5) | 1 (1.4) | 0 | 2 (1.0) |  |
| GR007 - Evangelismos General Hospital of Athens | 5 (7.4) | 3 (4.3) | 6 (9.0) | 14 (6.8) |  |
|  | | | | |  |
| **Mexico** | 1 (1.5) | 1 (1.4) | 2 (3.0) | 4 (2.0) |  |
| MX001 - Universidad Autonoma de Nuevo Leon | 1 (1.5) | 1 (1.4) | 2 (3.0) | 4 (2.0) |  |
|  |  |  |  |  |  |
| **Romania** | 18 (26.5) | 22 (31.4) | 21 (31.3) | 61 (29.8) |  |
| RO012 - Institutul National De Boli Infectioase | 8 (11.8) | 7 (10.0) | 7 (10.4) | 22 (10.7) |  |
| RO014 - Spitalul de Urgenta "Sf. Ioan Cel Nou" Suceava | 8 (11.8) | 9 (12.9) | 6 (9.0) | 23 (11.2) |  |
| RO016 - Spitalul de Boli Infect. si Tropical Dr.Victor Babes | 0 | 1 (1.4) | 0 | 1 (0.5) |  |
| RO017 - Spitalul De Boli "Sfanta Parascheva" | 2 (2.9) | 5 (7.1) | 8 (11.9) | 15 (7.3) |  |
|  |  |  |  |  |  |
| **Spain** | 40 (58.8) | 38 (54.3) | 36 (53.7) | 114 (55.6) |  |
| ES013 - Hospital Virgen del Rocio | 1 (1.5) | 1 (1.4) | 2 (3.0) | 4 (2.0) |  |
| ES015 - Hospital Universitari Germans Trias i Pujol | 0 | 0 | 1 (1.5) | 1 (0.5) |  |
| ES017 - Hospital Clinico San Carlos | 0 | 1 (1.4) | 2 (3.0) | 3 (1.5) |  |
| ES023 - Hospital Universitario HM Montepríncipe | 4 (5.9) | 2 (2.9) | 7 (10.4) | 13 (6.3) |  |
| ES024 - Hospital Universitario Ramón y Cajal | 1 (1.5) | 0 | 0 | 1 (0.5) |  |
| ES026 - Hospital Quirón Salud Madrid | 3 (4.4) | 3 (4.3) | 0 | 6 (2.9) |  |
| ES029 - H.U. Guadalajara | 1 (1.5) | 0 | 1 (1.5) | 2 (1.0) |  |
| ES031 - H.U. Salamanca | 0 | 1 (1.4) | 0 | 1 (0.5) |  |
| ES033 - Hospital Infanta Leonor | 1 (1.5) | 0 | 0 | 1 (0.5) |  |
| ES034 - Hospital Álvaro Cunqueiro | 0 | 0 | 1 (1.5) | 1 (0.5) |  |
| ES035 - Instituto de Investigación Sanitaria Valdecilla | 1 (1.5) | 1 (1.4) | 2 (3.0) | 4 (2.0) |  |
| ES036 - Hospital General Universitario de Alicante | 2 (2.9) | 1 (1.4) | 1 (1.5) | 4 (2.0) |  |
| ES037 - Hospital Universitario Madrid Sanchinarro | 2 (2.9) | 4 (5.7) | 3 (4.5) | 9 (4.4) |  |
| ES038 - Hospital Universitari de Bellvitge IDIBELL | 0 | 1 (1.4) | 0 | 1 (0.5) |  |
| ES040 - Hospital Enfermera Isabel Zendal | 22 (32.4) | 23 (32.9) | 14 (20.9) | 59 (28.8) |  |
| ES050 - Hospital Jerez de la Frontera | 0 | 0 | 1 (1.5) | 1 (0.5) |  |

**Supplementary Table 2:** Active Medical History by Grouped Preferred Term (≥ 5% of Patients in Any Treatment Arm) (Intent-to-treat Population)

| **Grouped Preferred Term^a^** | **Plitidepsin 2.5 mg**  **(N = 68)** | | **Plitidepsin 1.5 mg (N = 70)** | | **Control Arm (N = 67)** | | **Total (N = 205)** | |
| --- | --- | --- | --- | --- | --- | --- | --- | --- |
|  | N | % | N | % | N | % | N | **%** |
| Number of patients with at least 1 active medical history record^b^ | 58 | (85.3) | 53 | (75.7) | 53 | (79.1) | 164 | (80.0) |
| Hypertension | 30 | 44.1 | 27 | 38.6 | 25 | 37.3 | 82 | 40.0 |
| Diabetes mellitus^c^ | 12 | 17.6 | 14 | 20.0 | 17 | 25.4 | 43 | 21.0 |
| Obesity | 7 | 10.3 | 10 | 14.3 | 20 | 29.9 | 37 | 18.0 |
| Dyslipidemia | 12 | 17.6 | 11 | 15.7 | 9 | 13.4 | 32 | 15.6 |
| Menopause | 7 | 10.3 | 4 | 5.7 | 5 | 7.5 | 16 | 7.8 |
| Asthma | 4 | 5.9 | 7 | 10.0 | 4 | 6.0 | 15 | 7.3 |
| Constipation | 3 | 4.4 | 6 | 8.6 | 5 | 7.5 | 14 | 6.8 |
| Hypothyroidism | 2 | 2.9 | 5 | 7.1 | 6 | 9.0 | 13 | 6.3 |
| Benign prostatic hyperplasia | 4 | 5.9 | 4 | 5.7 | 5 | 7.5 | 13 | 6.3 |
| Insomnia | 2 | 2.9 | 5 | 7.1 | 4 | 6.0 | 11 | 5.4 |
| Anxiety | 1 | 1.5 | 4 | 5.7 | 4 | 6.0 | 9 | 4.4 |
| Sleep apnea syndrome | 3 | 4.4 | 1 | 1.4 | 4 | 6.0 | 8 | 3.9 |
| Seasonal allergy | - | - | 5 | 7.1 | 2 | 3.0 | 7 | 3.4 |
| Drug hypersensitivity | 1 | 1.5 | 1 | 1.4 | 4 | 6.0 | 6 | 2.9 |
| Chronic obstructive pulmonary disease | 1 | 1.5 | 1 | 1.4 | 4 | 6.0 | 6 | 2.9 |
| Rhinitis allergic | 1 | 1.5 | 4 | 5.7 | 1 | 1.5 | 6 | 2.9 |
| Tobacco user | 4 | 5.9 | - | - | 2 | 3.0 | 6 | 2.9 |
| Hemorrhoids | 4 | 5.9 | 1 | 1.4 | - | - | 5 | 2.4 |
| Peripheral venous disease | - | - | - | - | 4 | 6.0 | 4 | 2.0 |

Abbreviations: eCRF = electronic case report form; N = number of patients in analysis set; n = number of patients with data available; % = percentages are calculated based on N as the denominator.

^a^ Within a system organ class, a patient may have reported more than 1 grouped preferred term. Patients are counted once for each grouped preferred term and each system organ class.

^b^ Active medical conditions are those ticked on eCRF as ongoing (with or without treatment).

^c^ Includes diabetes mellitus and type 2 diabetes mellitus.

**Supplementary Table 3:** Baseline patient and disease characteristics of the intention-to-treat population by antiviral administration.

| **Characteristic Statistics** | **Remdesivir (N = 28)** | **Favipiravir (N = 8)** | **None (N = 31)** | **Control Arm (N = 67)** |
| --- | --- | --- | --- | --- |
| Age (years) |  |  |  |  |
| n | 28 | 8 | 31 | 67 |
| Mean (SD) | 60.1 (15.4) | 62 (14.6) | 57.9 (15.1) | 59.3 (15.0) |
| Age group (years), n (%) |  |  |  |  |
| ≥18 to 64 | 15 (53.6) | 3 (37.5) | 20 (64.5) | 38 (56.7) |
| ≥65 to 74 | 6 (21.4%) | 3 (37.5) | 6 (19.4) | 15 (22.4) |
| ≥75 | 7 (25) | 2 (25) | 5 (16.1) | 14 (20.9) |
| Sex, n (%) |  |  |  |  |
| Male | 15 (53.6) | 6 (75) | 21 (67.7) | 42 (62.7) |
| Female | 13 (46.4) | 2 (25) | 10 (32.3) | 25 (37.3) |
| Race, n (%)^a^ |  |  |  |  |
| White | 26 (92.9) | 8 (100) | 30 (96.8) | 64 (95.5) |
| Multiple | 2 (7.1) | 0 (0) | 1 (3.2) | 3 (4.5) |
| Body mass index at screening (kg/m^2^)^b^ | |  |  |  |
| n | 28 | 8 | 29 | 65 |
| Mean (SD) | 30.4 (6.4) | 28.8 (4.6) | 30.5 (6.3) | 30.3 (6.1) |
| Body mass index group at screening (kg/m^2^)^b^, n (%) | | |  |  |
| ≥18.5 and <25 | 5 (17.9) | 2 (25) | 3 (10.3) | 10 (14.9) |
| ≥25 and <30 | 11 (39.3) | 2 (25) | 13 (44.8) | 26 (38.8) |
| ≥30 and <35 | 5 (17.9) | 3 (37.5) | 9 (31) | 17 (25.4) |
| ≥35 and <40 | 5 (17.9) | 1 (12.5) | 2 (6.9) | 8 (11.9) |
| ≥40 | 2 (7.1) | 0 (0) | 2 (6.9) | 4 (6.0) |
| Missing | - | - | 2 (6.9) | 2 (3.0) |
| Chest imaging at enrolment, n (%) | | | |  |
| Pulmonary infiltrates | 15 (53.6) | 3 (37.5) | 25 (80.6) | 43 (64.2) |
| Bilateral pneumonia | 15 (53.6) | 1 (12.5) | 3 (9.7) | 19 (28.4) |
| Periods of inclusion, n (%) | | |  |  |
| Beginning of accrual – August 2021 | 0 (0) | 0 (0) | 2 (6.5) | 2 (3.0) |
| September 2021 - March 2022 | 19 (67.9) | 8 (100) | 24 (77.4) | 51 (76.1) |
| April 2022 - End of accrual | 9 (32.1) | 0 (0) | 5 (16.1) | 14 (20.9) |
| Time from symptom onset to treatment initiation | | |  |  |
| n | 28 | 8 | 29 | 65 |
| Mean (SD) | 5 (2) | 6.1 (2.2) | 6 (2.6) | 5.6 (2.3) |
| Respiration rate (breaths/min) at screening | | |  |  |
| n | 28 | 8 | 29 | 65 |
| Mean (SD) | 20.5 (2.8) | 21.9 (2.1) | 18.7 (3.7) | 19.9 (3.3) |
| Oxygen saturation (%) at screening^c^ | | |  |  |
| n | 28 | 8 | 31 | 67 |
| Mean (SD) | 95.7 (2) | 97.5 (0.8) | 96.8 (1.4) | 96.4 (1.7) |
| Fraction of inspired oxygen (%) at screening | | | | |
| n | 28 | 8 | 31 | 67 |
| Mean (SD) | 28.4 (14.3) | 29 (2.8) | 27.3 (3.7) | 27.9 (9.5) |
| Vaccination status, n (%) | | |  |  |
| Fully vaccinated | 13 (46.4) | 1 (12.5) | 18 (58.1) | 32 (47.8) |
| Non-fully vaccinated | 2 (7.1) | 0 (0) | 2 (6.5) | 4 (6.0) |
| Not vaccinated | 13 (46.4) | 7 (87.5) | 11 (35.5) | 31 (46.3) |
| S1 spike protein IgG at Day 1 | | |  |  |
| n | 28 | 7 | 24 | 59 |
| Negative | 13 (46.4%) | 3 (42.9%) | 8 (33.3%) | 24 (40.7) |
| Positive | 15 (53.6%) | 4 (57.1%) | 16 (66.7%) | 35 (59.3) |
| SARS-CoV-2 viral load at Day 1 (log_10_ copies/mL)^d^ | | | |  |
| n | 26 | 7 | 22 | 55 |
| Mean (SD) | 5.1 (2.2) | 4.2 (2.6) | 5.2 (2.2) | 5.1 (2.2) |

Abbreviations: n = number of patients with data available; N = number of patients in analysis set; SD = standard deviation; % = percentages are calculated based on N as the denominator.

^a^ Patients with more than 1 race reported were included in the multiple categories.

^b^ Body mass index (kg/m^2^) = weight (kg)/height (m^2^).

^c^ O_2_ at baseline estimated at ambient air with a correction for altitude. The specific modality (mask or nasal prongs) was not collected.

^d^ Summary was based on full analysis set.

**Supplementary Table 4:** Summary of Secondary Endpoints

| Variable | Plitidepsin 2.5 mg | Plitidepsin 1.5 mg | Control Arm | |
| --- | --- | --- | --- | --- |
| **Time to sustained hospital discharge** | **(N = 68)** | **(N = 70)** | **(N = 67)** | |
| Patients who reported sustained hospital discharge, n (%) | 55 (80.9) | 61 (87.1) | 59 (88.1) | |
| Time to sustained hospital discharge (days) 95% CI^a^ |  |  |  | |
| Median | 7 (7, 9) | 7 (NE, NE) | 7 (7, 9) | |
| Stratified log‑rank test |  |  |  | |
| 2‑sided p‑value^b^ | 0.59 | 0.34 |  | |
| 2‑sided p‑value adjusted for multiplicity^b,c^ | NA | NA |  | |
| Supportive analysis ‑ stratified Cox regression model |  |  |  | |
| Hazard ratio^d,e^ | 0.948 | 1.18 |  | |
| 95% CI for Hazard ratio^d,e^ | 0.655 – 1.37 | 0.827 – 1.70 |  | |
| 2‑sided p‑value^d^ | 0.78 | 0.35 |  | |
| **11‑point WHO Clinical Progression Scale  on Day 8** | **(N = 63)** | **(N = 67)** | **(N = 65)** | |
| 11-category WHO Clinical Progression Scale | |  |  | |
| Clinical Status [0 – 2] at Day 8 | 38 (60.3) | 42 (62.7) | 34 (52.3) | |
| 11‑category WHO Clinical Progression Scale on Day 8, n (%) (95% CI)f | | |  | |
| 0 = uninfected, no viral RNA detected | 6 (9.5)  (3.22–25.0) | 10 (14.9)  (6.43–31.0) | 3 (4.6)  (1.04–18.2) | |
| 1 = asymptomatic, viral RNA detected | 12 (19.0)  (8.88–36.2) | 15 (22.4)  (11.4–39.3) | 12 (18.5)  (8.60–35.3) | |
| 2 = symptomatic, independent | 20 (31.7)  (18.0–49.6) | 17 (25.4)  (13.5–42.5) | 19 (29.2)  (16.2–46.8) | |
| 3 = symptomatic, assistance needed | 0 (0.0)  (0–11.3) | 1 (1.5)  (0.150–13.2) | 1 (1.5)  (0.155–13.6) | |
| 4 = hospitalized, no oxygen therapy | 4 (6.3)  (1.71–20.9) | 8 (11.9)  (4.64–27.4) | 7 (10.8)  (3.93–26.3) | |
| 5 = hospitalized, oxygen by mask or nasal prongs | 10 (15.9)  (6.85–32.6) | 8 (11.9)  (4.64–27.4) | 20 (30.8)  (17.4–48.4) | |
| 6 = hospitalized, oxygen by NIV or high flow | 5 (7.9)  (2.43–23.0) | 7 (10.4)  (3.81–25.6) | 1 (1.5)  (0.155–13.6) | |
| 7 = intubation and mechanical ventilation. pO2/FiO2 ≥150 or SpO2/FiO2 ≥200 | 4 (6.3)  (1.71–20.9) | 1 (1.5)  (0.150–13.2) | 2 (3.1)  (0.527–16.0) | |
| 8 = mechanical ventilation pO2/FiO2 <150 (SpO2/FiO2 <200) or vasopressors | 1 (1.6)  (0.160–14.0) | 0 (0.0)  (0–10.7) | 0 (0.0)  (0–11.0) | |
| 9 = mechanical ventilation pO2/FiO2 <150 and vasopressors, dialysis, or ECMO | 0 (0.0)  (0–11.3) | 0 (0.0)  (0–10.7) | 0 (0.0)  (0–11.0) | |
| 10 = dead | 1 (1.6)  (0.160–14.0) | 0 (0.0)  (0–10.7) | 0 (0.0)  (0–11.0) | |
| Adjusted odds ratio^e,g^ | 1.12 | 1.69 |  | |
| 95% CI for adjusted odds ratio^e,g^ | 0.604 – 2.06 | 0.920 – 3.11 |  | |
| 2‑sided p‑value^g^ | 0.7252 | 0.0910 |  | |
| **Total Duration of Advanced Oxygen Support** | **(N = 63)** | **(N = 67)** | **(N = 65)** | |
| Patients who required advanced oxygen support, n (%) | 13 (20.6) | 11 (16.4) | 11 (16.9) | |
| Duration of advanced oxygen support (days)^h^ |  |  |  | |
| n | 13 | 11 | 11 | |
| Mean (SD) | 12.2 (9.71) | 10.0 (8.21) | 8.3 (12.66) | |
| **Patients who Required Intensive Care Unit Admission per Period** | **(N = 63)** | **(N = 67)** | **(N = 65)** | |
| From Day 1 to Day 4 |  |  |  | |
| Require ICU admission, n (%) | 1 (1.6) | 1 (1.5) | 3 (4.6) | |
| From Day 1 to Day 8 |  |  |  | |
| Require ICU admission, n (%) | 7 (11.1) | 3 (4.5) | 4 (6.2) | |
| Adjusted odds ratio^e,i^ | 1.94 | 0.703 |  | |
| 95% CI for adjusted odds ratio^e,i^ | 0.536 – 7.04 | 0.150 – 3.30 |  | |
| 2‑sided p‑value^i^ | 0.3125 | 0.6545 |  | |
| From Day 1 to Day 15 |  |  |  | |
| Require ICU admission, n (%) | 7 (11.1) | 4 (6.0) | 4 (6.2) | |
| From Day 1 to Day 31 |  |  |  | |
| Require ICU admission, n (%) | 7 (11.1) | 5 (7.5) | 4 (6.2) | |
| **Change in SARS-CoV-2 Viral Load (log_10_ copies/ml) From Day 1 to Day 8** | **(N = 38)** | **(N = 47)** | **(N = 46)** |  |
| Median | -1.74 | -2.24 | -2.83 | |
| Q1, Q3 | -3.39, -0.36 | -3.52, 0.00 | -3.75, -0.25 | |
| LS mean difference vs control (SE)^e,j,k^ | 0.64 (0.338) | 0.48 (0.321) |  | |
| 2-sided p-value^j^ | 0.062 | 0.14 |  | |
| **Patients with Undetectable SARS-CoV-2 Viral Load on Day 8** | **(N = 63)** | **(N = 67)** | **(N = 65)** | |
| n (%) | 14 (22.2) | 17 (25.4) | 15 (23.1) | |
| Adjusted odds ratio^e^ | 1.0 | 1.17 |  | |
| 95% CI for adjusted odds ratio^e^ | 0.426 – 2.26 | 0.523 – 2.61 |  | |
| 2‑sided p‑value | 0.97 | 0.70 |  | |

**Abbreviations**: **CI** = confidence interval; **ECMO** = extracorporeal membrane oxygenation; **eCRF** = electronic case report form; **FiO2** = fraction of inspired oxygen; **ICU** = intensive care unit; n = number of patients in each category; **LS**= least square; **N** = number of patients in analysis set; **NA** = not applicable; **NE** = not estimated; **NIV** = noninvasive ventilation; **pO2** = partial pressure of oxygen; **RNA** = ribonucleic acid; **SD** = standard deviation; **SE** = standard error; **WHO** = World Health Organization; **%** = percentages are calculated based on N as the denominator.

^a^ Calculated using the Kaplan Meier estimator. Confidence intervals derived based on Brookmeyer Crowley method and using a log log transformation.

^b^ 2-sided p values calculated using a stratified log rank test, including the fixed effect of the treatment arm and levels of the randomization stratification factors, i.e., geographical region (Europe versus Rest of the World), Charlson comorbidity index (0 to 1 versus >1) and prebaseline Barthel index (≥90 versus <90) as derived using the eCRF data as covariates.

^c^ Stratified log rank test p values adjusted for multiplicity using the Hochberg step up procedure. Not applicable since primary efficacy endpoint of time to sustained withdrawal of oxygen supplementation was not significant for both doses in the ITT population.

^d^ Hazard ratio, 95% CI and 2-sided p values calculated using a Cox proportional hazards regression model, including the fixed effect of the treatment arm and levels of the randomization stratification factors, i.e., geographical region (Europe versus Rest of the World), Charlson comorbidity index (0 to 1 versus >1) and prebaseline Barthel index (≥90 versus <90) as derived using the eCRF data as covariates. Hazard ratio >1 favors a plitidepsin arm to be associated with a shorter time to sustained hospital discharge than control arm.

^e^ Statistics calculated relative to control arm.

^f^ Goodman 95% CI for multinomial proportions.

^g^ Adjusted odds ratio, 95% CI and 2-sided p values based on a proportional odds model with fixed effects of treatment arm and randomisation stratification factors, i.e., geographical region (Europe versus Rest of the World), Charlson Comorbidity index (0 to 1 versus >1) and prebaseline Barthel index (≥90 versus <90) as derived using the eCRF data as covariates.

^h^ Duration of advanced oxygen support (days) = (date of last advanced oxygen support required date of randomization) + 1 off advanced oxygen support days.

^i^ Adjusted odds ratio, 95% CI and 2-sided p values based on a stratified logistic regression model with fixed effects of treatment arm and randomization stratification factors, i.e., geographical region (Europe versus Rest of the World), Charlson Comorbidity index (0 to 1 versus >1) and prebaseline Barthel index (≥90 versus <90) as derived using the eCRF data as covariates.

^j^ LS means, SEs, 95% CI and p-values are from an analysis of covariance (ANCOVA) model with fixed effects of treatment group, continuous SARS-CoV-2 viral load at Day 1, and categorical randomisation stratification factors, ie, geographical region (Europe vs. Rest of the World), Charlson Comorbidity Index (0-1 vs. >1) and pre-baseline Barthel index (≥90 versus <90) as derived using the eCRF data as covariates.

^k^ Difference is calculated as LS Mean in Plitidepsin 2.5mg - LS Mean in Control Arm or LS Mean in Plitidepsin 1.5mg - LS Mean in Control Arm.

**Supplementary Table 5:** Summary of Adverse Events with >10% incidence in any treatment arm by antiviral administration

| **Adverse Events Category Adverse Events Type** | **Remdesivir**  **(N =28) n (%)** | **Favipiravir  (N = 8) n (%)** | **None (N =29) n (%)** | **Control Arm (N = 65) n (%)** |
| --- | --- | --- | --- | --- |
| **Any TEAE** | 14 (50.0) | 6 (75.0) | 20 (69.0) | 40 (61.5) |
| Grade ≥3 | 1 (3.6) | 2 (25.0) | 8 (27.6) | 11 (16.9) |
| **Any treatment‑related TEAE to any study treatment^a^** | **9 (32.1)** | **5 (62.5)** | **10 (34.5)** | **24 (36.9)** |
| Grade ≥3 | . | . | 3 (10.3) | 3 (4.6) |
| **Any serious TEAE** | 1 (3.6) | 1 (12.5) | 3 (10.3) | 5 (7.7) |
| Grade ≥3 | 1 (3.6) | 1 (12.5) | 3 (10.3) | 5 (7.7) |
| **System Organ Class Grouped Preferred Term^a^ (Any-cause, all grade TEAEs)** | | | | |
| Gastrointestinal disorders | 4 (14.3) | 2 (25.0) | 6 (20.7) | 12 (18.5) |
| Constipation | 2 (7.1) |  | 2 (6.9) | 4 (6.2) |
| Nausea |  |  | 1 (3.4) | 1 (1.5) |
| Diarrhea | 1 (3.6) | 1 (12.5) | 1 (3.4) | 3 (4.6) |
| General disorders and administration site conditions | 3 (10.7) | 2 (25.0) | 8 (27.6) | 13 (20.0) |
| Investigations | 6 (21.4) | 4 (50.0) | 9 (31.0) | 19 (29.2) |
| Serum ferritin abnormal |  |  | 4 (13.8) | 4 (6.2) |
| C‑reactive protein increased |  |  | 5 (17.2) | 5 (7.7) |
| Alanine aminotransferase increased |  | 3 (37.5) | 2 (6.9) | 5 (7.7) |
| Gamma‑glutamyltransferase increased |  | 1 (12.5) | 2 (6.9) | 3 (4.6) |
| Metabolism and nutrition disorders | 3 (10.7) |  | 9 (31.0) | 12 (18.5) |
| Hyperglycemia^b^ | 3 (10.7) |  | 7 (24.1) | 10 (15.4) |
| Musculoskeletal and connective tissue disorders | 1 (3.6) |  | 3 (10.3) | 4 (6.2) |
| Nervous system disorders |  | 1 (12.5) | 5 (17.2) | 6 (9.2) |
| Headache |  | 1 (12.5) | 4 (13.8) | 5 (7.7) |
| Psychiatric disorders | 2 (7.1) |  | 3 (10.3) | 5 (7.7) |
| Sleep disorder | 2 (7.1) |  | 3 (10.3) | 5 (7.7) |
| Respiratory, thoracic, and mediastinal disorders | 1 (3.6) | 2 (25.0) | 10 (34.5) | 13 (20.0) |
| Acute respiratory distress syndrome |  | 1 (12.5) | 5 (17.2) | 6 (9.2) |
| Vascular disorders |  | 2 (25.0) | 4 (13.8) | 6 (9.2) |
| Phlebitis |  |  | 2 (6.9) | 2 (3.1) |

Abbreviations: n = number of patients with the reported Adverse Event; N = number of patients in analysis set; TEAE = treatment‑emergent adverse event; % = percentages are calculated based on N as the denominator.

^a^ Within a system organ class, patients could have reported more than 1 grouped preferred term. Patients were counted once for each grouped preferred term and each system organ class.

^b^ Hyperglycemia (system organ class= ‘Metabolism and nutrition disorders’) also includes ‘Blood glucose increased’ (system organ class= ‘Investigations’)

**Supplementary Table 6:** Summary of Treatment-Related Adverse Events reported in > 1 patient or as grade ≥ 3 by antiviral administration.

| **System Organ Class** | | **Preferred Term^a^** | **Remdesivir (N=63)** | | | | **Favipiravir (N=67)** | | | | **None (N=67)** | | | | **Control Arm (N=65)** | | | |
| --- | --- | --- | --- | --- | --- | --- | --- | --- | --- | --- | --- | --- | --- | --- | --- | --- | --- | --- |
|  |  |  | **All** | | **Grade**≥ **3** | | **All** | | **Grade**≥ **3** | | **All** | | **Grade**≥ **3** | | **All** | | **Grade≥ 3** | |
|  |  |  | **n** | **%** | **n** | **%** | **n** | **%** | **n** | **%** | **n** | **%** | **n** | **%** | **n** | **%** | **n** | **%** |
| **Investigations** | **ALT increased** | |  |  |  |  | 3 | 37.5 |  |  |  |  |  |  | **3** | **4.6** | **.** | **.** |
|  | **AST increased** | |  |  |  |  | 4 | 50.0 |  |  |  |  |  |  | **4** | **6.2** | **.** | **.** |
|  | **CPK increased** | |  |  |  |  |  |  |  |  | 2 | 6.9 |  |  | **2** | **3.1** | **.** | **.** |
|  | **Blood LDH increased** | |  |  |  |  | 1 | 12.5 |  |  | 2 | 6.9 |  |  | **3** | **4.6** | **.** | **.** |
| **Metabolism and nutrition disorders** | **Hyperglycemia^b^** | | 3 | 10.7 |  |  |  |  |  |  | 5 | 17.2 |  |  | **8** | **12.3** | **3** | **4.6** |
| **Vascular disorders** | **Hypertension** | |  |  |  |  | 2 | 25.0 |  |  |  |  |  |  | **2** | **3.1** | **.** | **.** |

Abbreviations: n = number of patients with the reported Adverse Event; N = number of patients in analysis set; % = percentages are calculated based on N as the denominator.

^a^ Within a system organ class, patients could have reported more than 1 grouped preferred term. Patients were counted once for each grouped preferred term and each system organ class.

^b ‘^Hyperglycemia’ (system organ class= ‘Metabolism and nutrition disorders’) also includes ‘Blood glucose increased’ (system organ class= ‘Investigations’).

**Supplementary Figure 1.** Time to sustained hospital discharge.

**

**
