# Supplementary figures and images for: A Phase III Randomized Controlled Trial of Plitidepsin, a Marine-Derived Compound, in Hospitalized Adults With Moderate COVID-19
Source: Clin Infect Dis. 2024 Aug 26;79(4):910–9. doi: 10.1093/cid/ciae227 (PMC11478586; doi:10.1093/cid/ciae227)

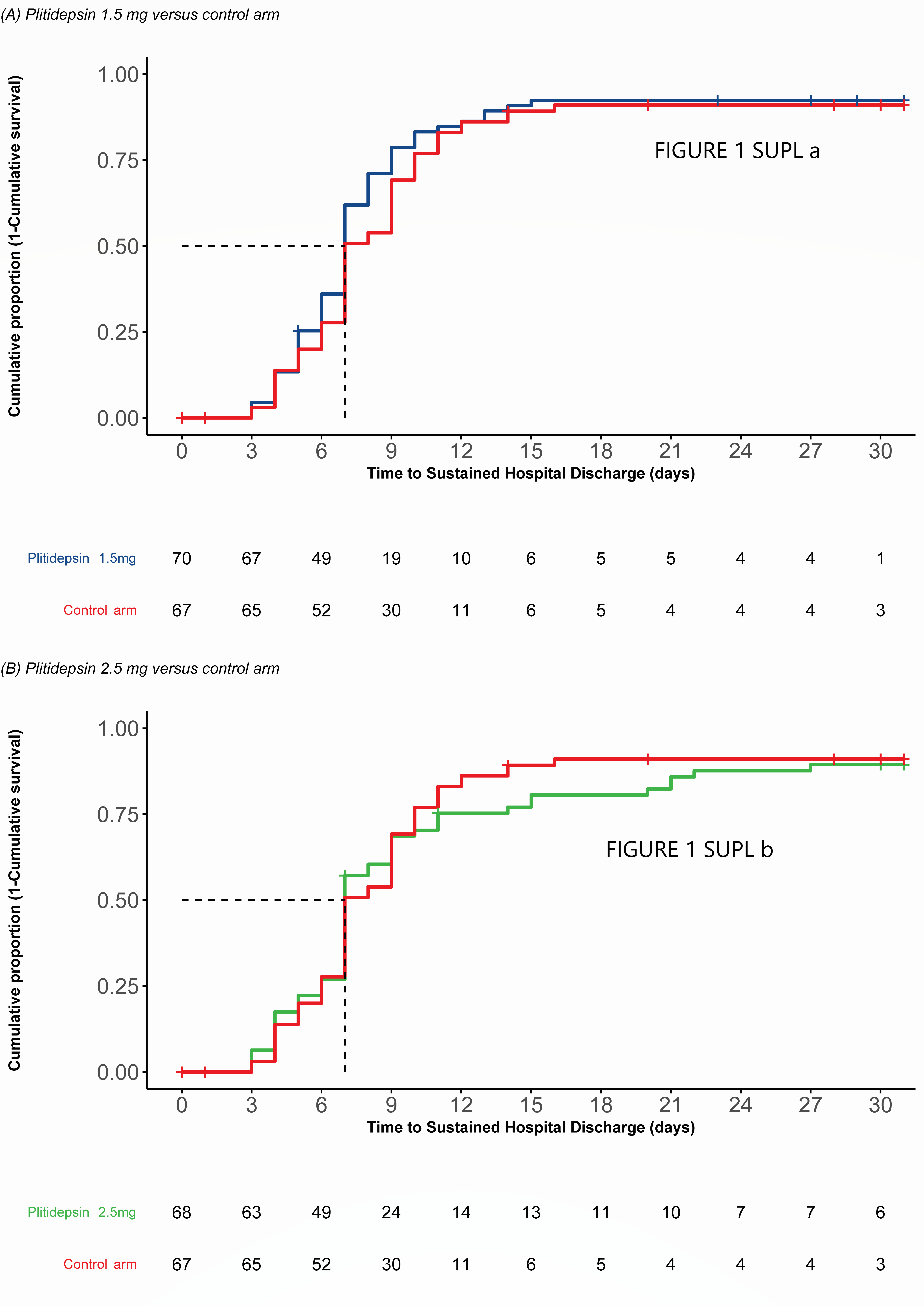

Supplement: ciae227_Supplementary_Data [file ciae227_supplementary_data.zip › Figure 1 Supplementary Multigraph.tiff]
